# An Enigmatic Cretaceous Beetle in Kachin Amber With Tentative Affinities to Pythidae (Coleoptera: Tenebrionoidea)

**DOI:** 10.1002/ece3.70615

**Published:** 2024-12-09

**Authors:** Yan‐Da Li, Darren A. Pollock, M. Andrew Johnston, Di‐Ying Huang, Chen‐Yang Cai

**Affiliations:** ^1^ State Key Laboratory of Palaeobiology and Stratigraphy Nanjing Institute of Geology and Palaeontology, Chinese Academy of Sciences Nanjing China; ^2^ Bristol Palaeobiology Group, School of Earth Sciences University of Bristol Bristol UK; ^3^ Department of Biology Eastern New Mexico University Portales New Mexico USA; ^4^ Department of Entomology Purdue University West Lafayette Indiana USA

**Keywords:** Cretaceous, fossil, Kachin amber, parsimony, phylogenetic analysis, Tenebrionoidea

## Abstract

Tenebrionoids are not uncommon in late Mesozoic ambers, but their paleodiversity remains poorly explored, partly due to the insufficiently defined families of the extant fauna. Here, we describe and illustrate a new tenebrionoid beetle from mid‐Cretaceous Kachin amber, *Glyphonotum hsiaoi* gen. et sp. nov., which exhibits an unusual form of pronotal sulcus and tarsi. Based on morphological comparison and phylogenetic analyses, we tentatively place *Glyphonotum* in the extant family Pythidae, representing the first Mesozoic fossil of this family.

## Graphical abstract

We describe and illustrate a new tenebrionoid beetle from mid‐Cretaceous Kachin amber, *Glyphonotum* *hsiaoi* gen. et sp. nov. Based on morphological comparison and phylogenetic analyses, we tentatively place *Glyphonotum* in the extant family Pythidae, representing the first Mesozoic fossil of this family.

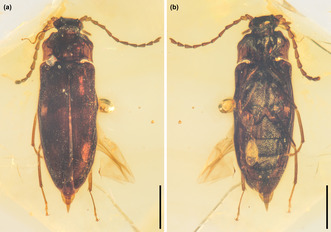

## 1 Introduction

The superfamily Tenebrionoidea stands out as one of the most diverse beetle groups, with around 50,000 described species. Tenebrionoidea currently include 29 extant families, plus a few groups with uncertain familial attribution (Bouchard et al. 2024). Tenebrionoids can be generally distinguished by their 5‐5‐4 tarsal formula in both sexes (although tarsomeres may be reduced in certain taxa) and the male genitalia with the phallobase dorsad or ventrad of the penis but not forming a ring around it (Lawrence, Pollock, and Ślipiński 2010). Earlier studies on tenebrionoid phylogenies based on a few gene markers yielded largely inconsistent interfamilial relationships (e.g., Gunter et al. 2014; Bocak et al. 2014; McKenna et al. 2015). Recent phylogenomic studies have begun to reach some consensus on the higher relationships within Tenebrionoidea (Zhang et al. 2018; McKenna et al. 2019; Cai et al. 2022). Ripiphoridae and Mordellidae (and possibly Stenotrachelidae) are the earliest diverging lineages, and the remaining families are divided into two major groups, one including Ischaliidae, Aderidae, Trictenotomidae, Scraptiidae, Mycteridae, Lagrioididae, Prostomidae, Oedemeridae, Boridae, Pythidae, Salpingidae, Pyrochroidae, Anthicidae, and Meloidae, and the other one including Zopheridae, Melandryidae, Ciidae, Synchroidae, Pterogeniidae, Tetratomidae, Archeocrypticidae, Mycetophagidae, Chalcodryidae, Promecheilidae, Ulodidae, and Tenebrionidae.

The earliest tenebrionoid fossils, suggested to be related to Rhipiphoridae and Mordellidae, were reported from the Middle Jurassic Haifanggou Formation (Daohugou, northeastern China) and the Upper Jurassic Karabastau Formation (Karatau, Kazakhstan) (Wang and Zhang 2011; Hsiao et al. 2017; Bao, Zhang, et al. 2019). Tenebrionidae were also known from the Karabastau Formation (Medvedev 1969; Nabozhenko 2019). Cretaceous tenebrionoids have been found in Yixian Formation, Lushangfen Formation (China), Kheta Formation, Turga Formation, Yakutian amber (Russia), Salignac amber, Charentese amber (France), New Jersey amber (USA), Lebanese amber, and Spanish amber, including members assigned to Ripiphoridae, Mordellidae, Scraptiidae, Melandryidae, Tetratomidae, Anthicidae, Aderidae, Oedemeridae, and Tenebrionidae (Wang and Zhang 2011: appendix 1; Kirejtshuk and Azar 2008, 2013; Kirejtshuk, Nabozhenko, and Nel 2011; Peris and Ruzzier 2013; Soriano et al. 2014; Nabozhenko et al. 2015; Chang et al. 2016; Peris 2017; Telnov et al. 2022). The greatest diversity of Mesozoic Tenebrionoidea has been documented in mid‐Cretaceous Kachin amber of northern Myanmar, including members assigned to Ripiphoridae (e.g., Batelka, Engel, and Prokop 2018, 2021; Cai, Yin, and Huang 2018), Mordellidae (e.g., Bao, Walczyńska, et al. 2019; Bao 2020), Ischaliidae (Telnov et al. 2023), Aderidae (Bao et al. 2022), Prostomidae (e.g., Li, Hsiao, Huang, et al. 2022), Oedemeridae (Vitali and Ellenberger 2019; Vitali and Legalov 2020), Salpingidae (Jiang, Liu, and Chen 2024), Meloidae (Poinar and Brown 2015), Zopheridae (e.g., Deng et al. 2017; Cheng et al. 2021; Li, Huang, and Cai 2021; Li, Jin, et al. 2024), Melandryidae (Tihelka, Huang, and Cai 2020; Li, Hsiao, Yoshitomi, et al. 2022), Tetratomidae (e.g., Cai, Hsiao, and Huang 2016; Yu et al. 2016; Hsiao 2020), and Tenebrionidae (Bao and Antunes‐Carvalho 2020). Tihelka et al. (2021) also reported the unusual *Kulindrobor* Tihelka et al. from Kachin amber, which cannot be easily assigned to any of the extant tenebrionoid families.

In this study, we describe a new fossil of Tenebrionoidea from Kachin amber. Based on the result of phylogenetic analyses, we tentatively assign it to the family Pythidae, representing the earliest fossil record of this family.

The Eocene *Pythoceropsis singularis* Wickham was described based on a ventral impression fossil from the Florissant formation, Colorado, USA (Wickham 1913). According to Wickham (1913: 21), “there is no doubt of the pythid affinities.” However, at the time of publication of that paper, the family concept of Pythidae was broader than it is at present, including taxa now considered to be part of the families Boridae, Salpingidae, and Mycteridae. In particular, the Pythini were thought to include, among a few other genera, *Pytho* Latreille, *Boros* Herbst, and *Lecontia* Champion (the latter two genera now firmly established in Boridae). Wickham compared the sculpturing and antennal structure of *Pythoceropsis* Wickham with all three extant genera, but based on the description and illustration of the fossil, attribution to Pythidae or Boridae is highly speculative.

## 2 Material and Methods

### 2.1 Material

**FIGURE 1:**
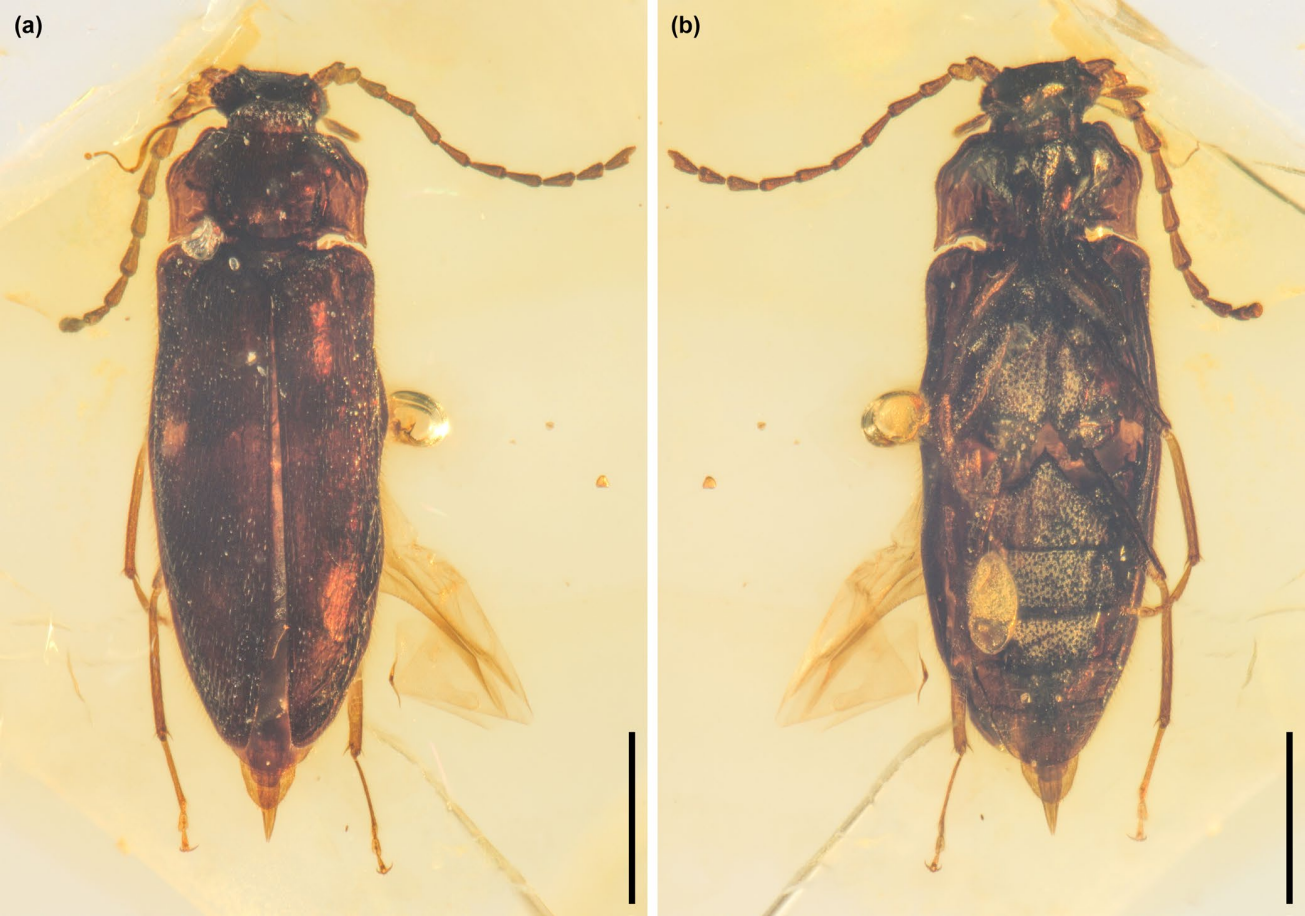
General habitus of *Glyphonotum hsiaoi* gen. et sp. nov., holotype, NIGP200729, under incident light. (a) Dorsal view; (b) Ventral view. Scale bars: 500 μm.

**FIGURE 2:**
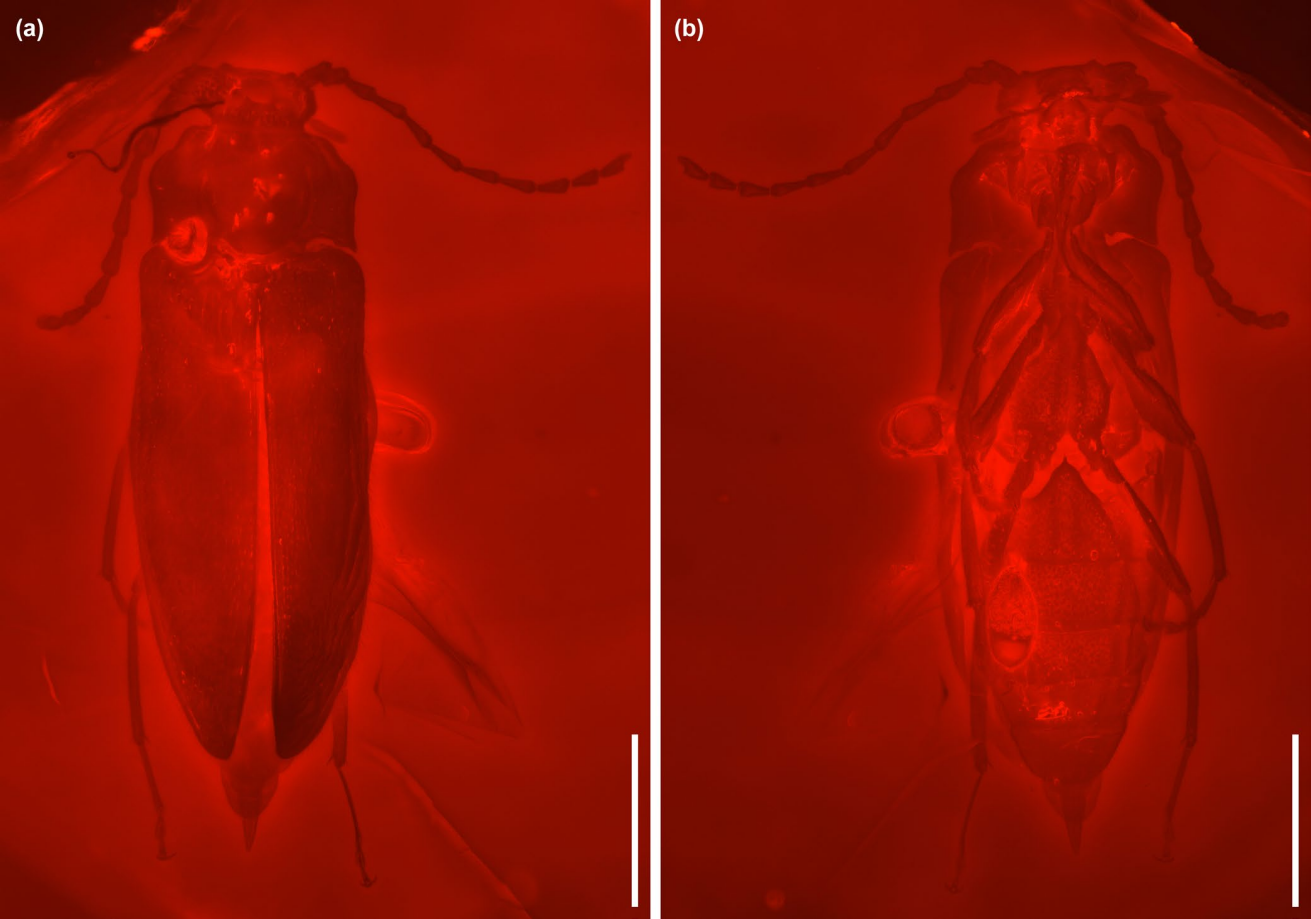
General habitus of *Glyphonotum hsiaoi* gen. et sp. nov., holotype, NIGP200729, under widefield fluorescence. (a) Dorsal view; (b) Ventral view. Scale bars: 500 μm.

**FIGURE 3:**
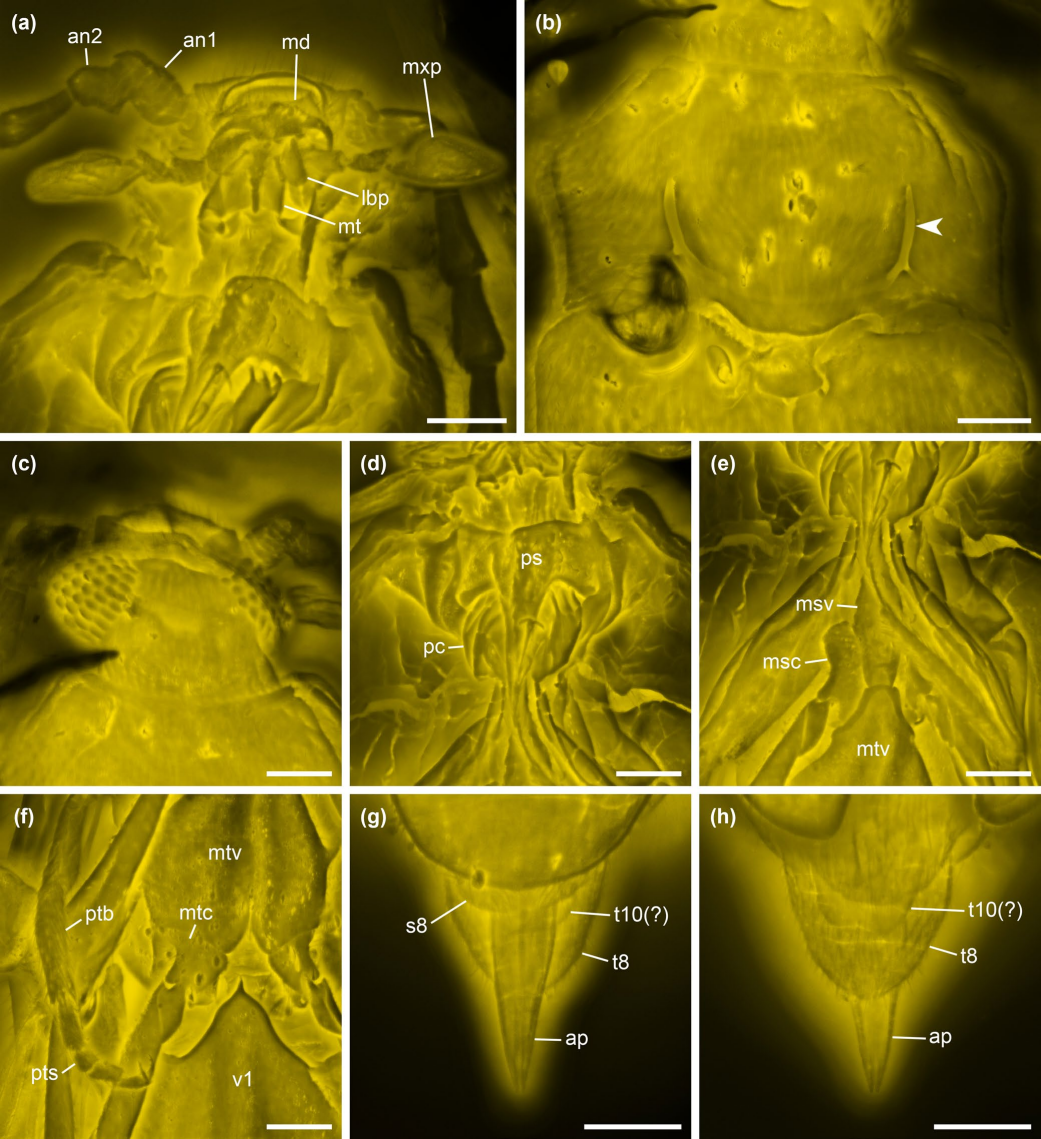
Details of *Glyphonotum hsiaoi* gen. et sp. nov., holotype, NIGP200729, under confocal microscopy. (a) Head, ventral view; (b) Prothorax, dorsal view, with arrowhead showing the U‐shaped sulci; (c) Head, dorsal view; (d) Prothorax, ventral view; (e) Mesothorax, ventral view; (f) Metathorax and abdominal base, ventral view; (g) Abdominal apex, ventral view; (h) Abdominal apex, dorsal view. an1–2, antennomeres 1–2; ap, apicale; lbp, labial palp; md, mandible; msc, mesocoxa; msv, mesoventrite; mt, mentum; mtc, metacoxa; mtv, metaventrite; mxp, maxillary palp; pc, procoxae; ps, prosternum; ptb, protibia; pts, protarsus; s8, sternite VIII; t8, tergite VIII; t10, tergite X; v1, ventrite 1. Scale bars: 100 μm.

**FIGURE 4:**
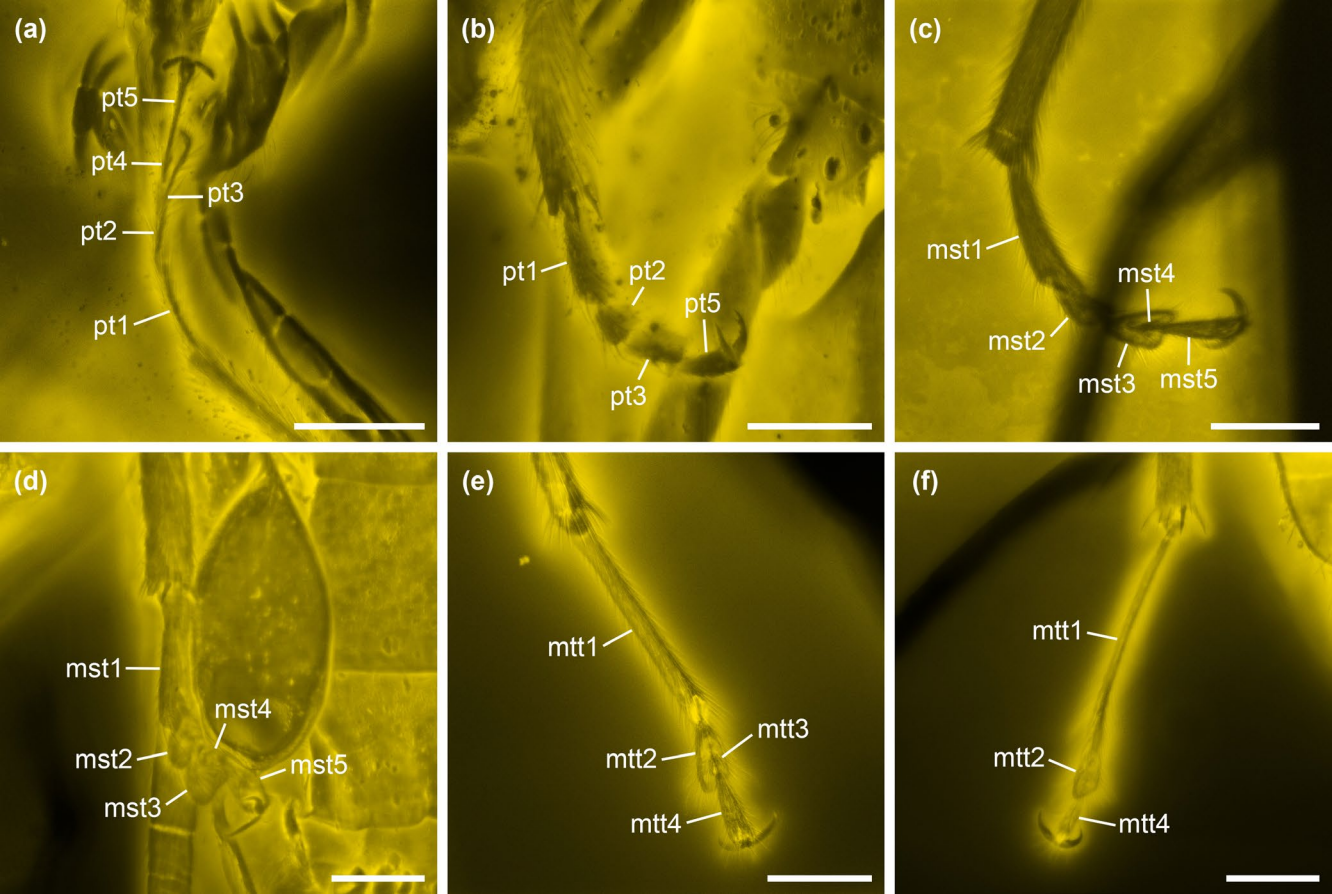
Details of *Glyphonotum hsiaoi* gen. et sp. nov., holotype, NIGP200729, under confocal microscopy. (a–b) Protarsus; (c–d) Mesotarsus; (e–f) Metatarsus. mst1–5, mesotarsomeres 1–5; mtt1–4, metatarsomeres 1–4; pt1–5, protarsomeres 1–5. Scale bars: 100 μm.

**FIGURE 5:**
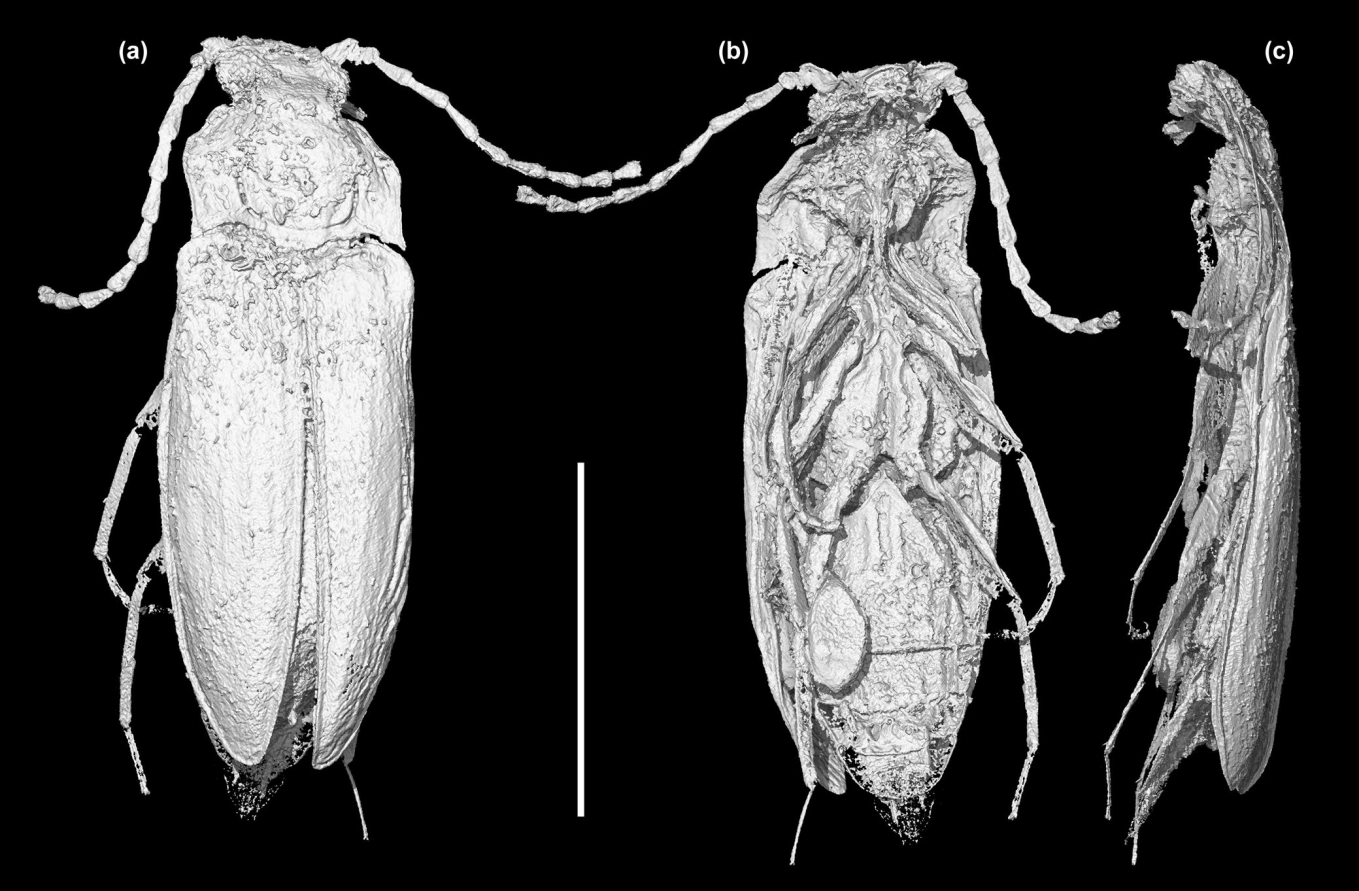
X‐ray microtomographic reconstruction of *Glyphonotum hsiaoi* gen. et sp. nov., holotype, NIGP200729. (a) Dorsal view; (b) Ventral view; (c) Lateral view. Scale bar: 1 mm.

The Kachin (Burmese) amber specimen studied herein (Figures 1, 2, 3, 4, 5) originated from amber mines near Noije Bum (26°20′ N, 96°36′ E), Hukawng Valley, Kachin State, northern Myanmar. The amber specimen is deposited in the Nanjing Institute of Geology and Paleontology (NIGP), Chinese Academy of Sciences, Nanjing, China. The amber piece was trimmed with a small table saw, ground with emery paper of different grit sizes, and finally polished with polishing powder.

### 2.2 Fossil Imaging

Photographs under incident light were taken with a Zeiss Discovery V20 stereo microscope. Widefield fluorescence images were captured with a Zeiss Axio Imager 2 light microscope combined with a fluorescence imaging system. Confocal images were obtained with a Zeiss LSM710 confocal laser scanning microscope using the 561 nm (DPSS 561‐10) laser excitation line (Fu et al. 2021). Images under incident light and widefield fluorescence were stacked mainly in Helicon Focus 7.0.2 or Zerene Stacker 1.04. Confocal images were stacked with Helicon Focus 7.0.2 and Adobe Photoshop CC. Microtomographic data were obtained with a Zeiss Xradia 520 Versa 3D X‐ray microscope at the micro‐CT laboratory of NIGP and analyzed in VGStudio MAX 2022.2. Scanning parameters were as follows: isotropic voxel size, 2.6143 μm; power, 3 W; acceleration voltage, 40 kV; exposure time, 4 s; projections, 3001. Images were further processed in Adobe Photoshop CC to adjust brightness and contrast.

### 2.3 Phylogenetic Analyses

To evaluate the systematic placement of the new fossil, we conducted constrained morphology‐based phylogenetic analyses under parsimony (e.g., Fikáček et al. 2020; Li, Liu, et al. 2023). The data matrix (File S1) was derived from the previously published dataset by Lawrence et al. (2011), and the character list can be found in Lawrence et al. (2011). The original matrix by Lawrence et al. (2011) had broad sampling among the whole Coleoptera, but in the present analysis we only selected the tenebrionoid genera and the lymexyloid outgroups.

In the constrained analyses, the relationships among extant taxa were fixed as the backbone tree according to previous molecular studies, and only the fossil was allowed to move freely. For the construction of backbone trees, the interfamilial relationships were mainly based on Cai et al. (2022) and McKenna et al. (2019); when the phylogenies by Cai et al. (2022) and McKenna et al. (2019) conflict with each other, we generally followed Cai et al. (2022), as the site‐heterogeneous model used there has been proven to consistently outperform the site‐homogeneous models as used by McKenna et al. (2019) (e.g., Lartillot, Brinkmann, and Philippe 2007; Tihelka et al. 2020; Li, Engel, et al. 2023); the relationships not covered by the above studies were mainly based on McKenna et al. (2015).

The analyses (File S2) were performed under both equal and implied weights, using R 4.1.0 (R Core Team 2021) and the R package TreeSearch 1.3.1 (Smith 2023). The concavity constant in the weighted analysis was set to 12, following the suggestion by Goloboff, Torres, and Arias (2018) and Smith (2019). To inspect both the optimal and suboptimal placements of the fossil, the parsimony scores of the trees with alternative placements of the fossil were mapped to the corresponding branches of the backbone tree (Li, Liu, et al. 2024; Li, Kolibáč, et al. 2024; Li, Tomaszewska, et al. 2024). The results were visualized with the R package ggtree 6.5.2 (Yu et al. 2017; Yu 2020) and graphically edited with Adobe Illustrator CC 2017.

## 3 Systematic Paleontology

Order Coleoptera Linnaeus, 1758

Suborder Polyphaga Emery, 1886

Superfamily Tenebrionoidea Latreille, 1802

Family (?) Pythidae Solier, 1834

### 3.1 Genus *Glyphonotum* gen. nov.

**Type species**. *Glyphonotum hsiaoi* sp. nov.

**Etymology**. The generic name is formed based on the Greek “*glypho*,” carving, and “*noton*,” notum, referring to the distinct sulcus on the pronotal disc. The name is neuter in gender.

**Diagnosis**. Body elongate. Antennae filiform. Mandibles small (Figure 3a). Pronotum with complete lateral carinae; disc with U‐shaped sulcus (Figure 3b). Prosternal process complete. Procoxal cavities narrowly separated, externally open (Figure 3d). Procoxae not strongly projecting (Figure 3d). Tarsi 5‐5‐4; all tarsi with penultimate tarsomere reduced and antepenultimate tarsomere lobed beneath (Figure 4). Apicale apically narrowly notched, without paired accessory lobes (Figure 3g,h).

### 3.2 *Glyphonotum hsiaoi* sp. nov.

**Material**. Holotype, NIGP200729, male (Figures 1, 2, 3, 4, 5).

**Etymology**. The species is named after Dr. Yun Hsiao, a young coleopterist who has contributed to the taxonomy of several tenebrionoid families.

**Locality and horizon**. Amber mine located near Noije Bum Village, Tanai Township, Myitkyina District, Kachin State, Myanmar; unnamed horizon, mid‐Cretaceous, Upper Albian to Lower Cenomanian.

**Diagnosis**. As for the genus.

**Description**. Body elongate, flattened, about 2.1 mm long, 0.7 mm wide; surface with fine pubescence.

Head narrowed immediately behind eyes; temples absent. Compound eyes large and protuberant, relatively coarsely faceted; interfacetal setae present. Antennal socket distinctly raised laterally. Antennae 11‐segmented, elongate and distinctly filiform, without distinct club; antennomere 2 attached to antennomere 1 subapically. Mandibles small, strongly curved mesally. Maxillary palps 4‐segmented; terminal palpomere securiform. Mentum likely with median longitudinal carina (but also potentially an artifact of preservation).

Pronotum transverse, widest posteriorly, at base slightly narrower than elytral bases; lateral pronotal carinae complete, simple; lateral margins subparallel in posterior half, anteriorly converging in anterior half; anterior edge straight; posterior edge bisinuate; anterior angles not produced; posterior angles acute; U‐shaped sulcus present on pronotal disc, anterolaterally deep and distinct, posteromedially shallow and indistinct, with narrow and shallow collateral branch at each posterolateral corner. Prosternum in front of coxae well‐developed. Prosternal process elongate, gradually narrowing posteriorly, extending well beyond posterior edge of procoxae. Procoxal cavities narrowly separated, externally open. Procoxae slightly projecting. Protrochantins not observed.

Scutellar shield anteriorly not abruptly elevated, posteriorly broadly rounded. Elytra elongate, about 2.1 times as long as combined width, subparallel‐sided anteriorly and tapered posteriorly, covering almost all abdomen; elytral apices smoothly rounded; humeri distinct; disc flat, smooth, with scattered punctures; epipleura well‐developed anteriorly, posteriorly incomplete. Hind wings present. Mesocoxal cavities narrowly separated. Mesocoxae slightly projecting. Metaventrite with short externally visible discrimen; katepisternal suture absent. Metacoxae narrowly separated, laterally not meeting elytra; metacoxal plates absent.

Legs long. Trochanters heteromeroid. Tibiae and tarsi more densely setose than femora; all tibiae with paired apical spurs. Tarsi 5‐5‐4; pro‐ and mesotarsi with tarsomere 1 elongate and simple, tarsomere 2 weakly lobed, tarsomere 3 distinctly lobed beneath, tarsomere 4 reduced; metatarsi with tarsomere 1 distinctly elongate, tarsomere 2 distinctly lobed beneath, tarsomere 3 reduced. Pretarsal claws non‐dentate.

Abdomen with five ventrites; ventrite 1 with acute intercoxal process. Apicale narrowly notched apically (parameres not completely fused apically), without paired accessory lobes.

## 4 Discussion

Several groups within Tenebrionoidea can be easily ruled out as close relatives of *Glyphonotum* gen. nov. based on prothoracic characters (Lawrence 1977). *Glyphonotum* has externally open procoxal cavities, which separate it from Prostomidae, Archeocrypticidae, Chalcodryidae, Promecheilidae, Ulodidae, and Tenebrionidae (except for Zolodininae, Nilioninae, Kuhitangiinae, a few Pimeliinae, and some Cteniopodini; Matthews et al. 2010; Nabozhenko and Sadeghi 2017), where the procoxal cavities are almost always externally closed. The procoxae of *Glyphonotum* are not strongly projecting, which differentiates it from Stenotrachelidae, Ripiphoridae, Mordellidae, Ischaliidae, Aderidae, Scraptiidae, Lagriodidae, Oedemeridae, Pyrochroidae, Meloidae, Anthicidae, and Afreminae, in which the procoxae usually project well below the prosternum and are often contiguous or subcontiguous. *Glyphonotum* has complete lateral pronotal carinae, which differentiate it from Mycteridae and the *Rhizonium* group (and many of the families with projecting procoxae listed above), where the lateral pronotal carinae are incomplete or absent.

*Glyphonotum* could be further excluded from the following families based on certain characters. Mycetophagidae and Ciidae have clavate to capitate antennae and reduced (4‐4‐4, 3‐4‐4 or 3‐3‐3) tarsomeres (Lawrence et al. 2014; Lawrence 2016, 2019) (antennae filiform and tarsi 5‐5‐4 in *Glyphonotum*). Boridae have relatively short and usually capitate antennae (Lawrence and Pollock 1994). Trictenotomidae have very prominent mandibles and a head capsule that is unconstricted and rather broad behind eyes (e.g., Telnov and Drumont 2020) (mandibles small and head narrowed behind eyes in *Glyphonotum*). Pterogeniidae have a shorter body (about 1.7–2.1 times as long as wide) and usually an unconstricted head behind eyes (except for the unusual males of *Pterogenius* Candèze, which have a strongly expanded head) (Lawrence 1977; Burckhardt and Löbl 1992) (body about 3 times as long as wide in *Glyphonotum*).

**FIGURE 6:**
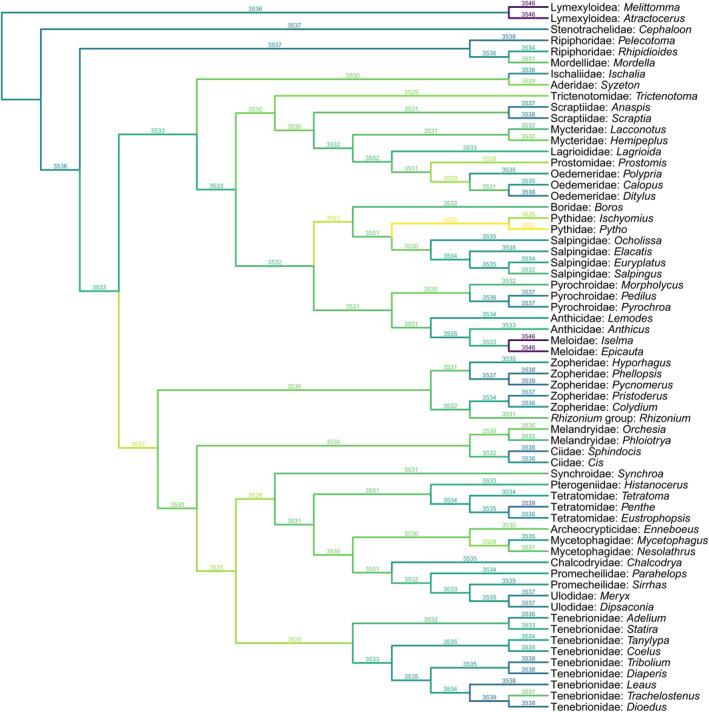
Constrained parsimony analysis under equal weights, showing alternative placements of *Glyphonotum* gen. nov. The score above each branch represents the parsimony score of the topology in which *Glyphonotum* is inserted to that branch.

**FIGURE 7:**
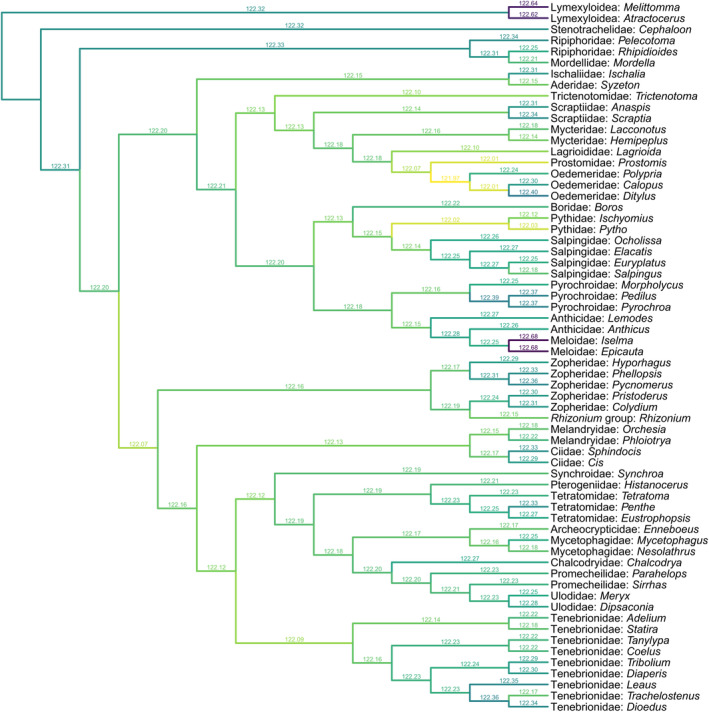
Constrained parsimony analysis under implied weights, showing alternative placements of *Glyphonotum* gen. nov. The score above each branch represents the parsimony score of the topology in which *Glyphonotum* is inserted to that branch.

The remaining tenebrionoid groups, including Pythidae, Salpingidae, Zopheridae, Melandryidae, Synchroidae, and Tetratomidae, are more difficult to separate from *Glyphonotum* by a few characters. In this case, the phylogenetic analyses may provide some helpful information on the position of *Glyphonotum*. Under equal weighting, the tree requires the minimum number of evolutionary steps when *Glyphonotum* is placed in Pythidae (Figure 6). Under implied weighting, the tree appears to be most parsimonious when *Glyphonotum* is placed in Pythidae or the group of Prostomidae + Oedemeridae, with the Prostomidae + Oedemeridae hypothesis having a slightly better (lower) parsimony score (Figure 7). We believe that *Glyphonotum* is unlikely to belong to Prostomidae or Oedemeridae, as they clearly differ from *Glyphonotum* in the prothoracic characters discussed above. Furthermore, the support for *Glyphonotum* in Prostomidae + Oedemeridae might be caused by the possibly artificial grouping of Prostomidae and Oedemeridae. The position of Prostomidae in the backbone tree was not determined by the phylogenomic analyses of Cai et al. (2022) or McKenna et al. (2019). Instead, it was based on the eight‐gene phylogeny by McKenna et al. (2015), which is much less reliable. Actually, in other molecular studies based on a few genes, Prostomidae have been variably grouped with Melandryidae, Pyrochroidae, Anthicidae, Salpingidae, or Mycteridae (Bocak et al. 2014; Gunter et al. 2014; Batelka, Kundrata, and Bocak 2016; Liu et al. 2023).

Therefore, we suggest that *Glyphonotum* most likely belongs in Pythidae. Pythidae are a small family, as currently defined including only *Pytho*, *Priognathus* LeConte, *Anaplopus* Blackburn, *Sphalma* Horn, *Ischyomius* Chevrolat, and possibly also *Osphyoplesius* Winkler (Pollock and Lawrence 1995; Pollock 2010; Magrini and Uliana 2015; Pollock and McClarin 2020). *Trimitomerus* Horn, once included in Pythidae, has been moved into Boridae (Kanda 2017; Lawrence et al. 2022). Although in most pythids, the prosternal process is incomplete with procoxal cavities contiguous, and in some of them the lateral pronotal carinae are absent or weakly developed, the genus *Ischyomius* has a well‐developed prosternal process and distinct lateral pronotal carinae (Pollock 1998, 2007, 2009). Filiform antennae are seen in some *Ischyomius* and *Anaplopus*. The overall appearance of *Glyphonotum* is also similar to some *Ischyomius* (e.g., Pollock 1998: figure 1E). The apicale of *Glyphonotum* lacks the paired accessory lobes characteristic of Pythidae, Scraptiidae, and most Salpingidae (e.g., Pollock 1991: figure 3), which might represent a secondary loss in *Glyphonotum*. A secondary loss of the accessory lobes is also reported for some Salpingidae taxa (Spilman 1952) and are reduced in size in some Scraptiidae (Johnston, Naczi, and Gimmel 2024) where they may not be visible in the position of the one fossil specimen studied here.

It should nevertheless be noted that the taxon sampling for the phylogenetic analyses in Lawrence et al. (2011) was not complete. The morphologically and phylogenetically heterogeneous Melandryidae are represented by only two genera in Orchesiini and Dircaeini in the analyses. Actually, many characters of *Glyphonotum* can be found in some lineages of Melandryidae (Nikitsky and Pollock 2010). For example, many Zilorini and Osphyinae have complete lateral pronotal carinae visible for their entire length from above; Osphyinae additionally have a head capsule narrowed behind eyes; and members of Orchesiini have a complete prosternal process and not or slightly projecting procoxae. However, none of them possess these characters simultaneously. Tetratomidae, once included in Melandryidae, are also a morphologically heterogeneous group (Nikitsky 1998), which is not adequately represented in the phylogenetic analyses. The filiform antennae can be found in Penthinae and Hallomeninae. A transverse sulcus connecting the basal pronotal pits is developed in a few tetratomid species, somewhat similar to the sulcus in *Glyphonotum*. However, Tetratomidae always have simple narrow tarsi and usually have a head capsule that is unconstricted and broad behind the eyes. Thus, we think *Glyphonotum* is less likely to be a member of Melandryidae or Tetratomidae, although this possibility cannot be completely ruled out.

The tarsal lobes of *Glyphonotum* are somewhat unusual in Tenebrionoidea. Tarsal lobes may be found in many tenebrionoid groups, i.e., Mordellidae, Ischaliidae, Aderidae, Scraptiidae, Mycteridae, Lagriodidae, Oedemeridae, Pythidae, Pyrochroidae, Anthicidae, Zopheridae: Colydiinae, Melandryidae, and Tenebrionidae. In most of these groups, when tarsal lobes are present, usually only the penultimate tarsomere is lobed ventrally. In Colydiinae, the first one or two tarsomeres may be enlarged and lobed, but their tarsi are generally 4‐4‐4 (Ślipiński and Lawrence 1999; Ivie et al. 2016). Anaspidini (Scraptiidae) have a lobed antepenultimate tarsomere in the fore and mid legs, but the metatarsomeres are all simple and not lobed (Franciscolo 1964, 1972; Johnston, Naczi, and Gimmel 2024). The only genus in Pythidae with tarsal lobes, *Ischyomius*, has ventral lobes on all tarsomeres except the apical one (Pollock 1998). The state similar to *Glyphonotum*, i.e., all tarsi with penultimate tarsomere reduced and antepenultimate tarsomere lobed beneath, is known only in Aderidae and *Heterotarsus* (Tenebrionidae) (e.g., Kaszab 1976; Werner 1990; Grzymala and Leschen 2020).

The distinct sulcus on the pronotal disc is another unusual character of *Glyphonotum*. Although basolateral impressions are common in Tenebrionoidea, such a sharply defined, long, and narrow sulcus is extremely rare. A somewhat similar sulcus may be variably distinctly developed in some Anthicidae, but anthicids have no lateral pronotal carinae, and the sulcus usually extends to the ventral side of the prothorax (e.g., Werner and Chandler 1995; Lawrence et al. 2011: figure 22D; Kejval and Chandler 2020).

## Author Contributions

**Yan‐Da Li:** conceptualization (equal), data curation (equal), formal analysis (equal), investigation (equal), visualization (equal), writing – original draft (equal), writing – review and editing (equal). **Darren A. Pollock:** investigation (equal), writing – review and editing (equal). **M. Andrew Johnston:** investigation (equal), writing – review and editing (equal). **Di‐Ying Huang:** funding acquisition (equal), investigation (equal), writing – review and editing (equal). **Chen‐Yang Cai:** conceptualization (equal), funding acquisition (equal), investigation (equal), supervision (equal), writing – review and editing (equal).

## Conflicts of Interest

The authors declare no conflicts of interest.

## Supporting information


**File S1** Morphological data matrix for phylogenetic analyses (character list in Lawrence et al., 2011).

**File S2** R script for evaluating the alternative placements of *Glyphonotum*.

## Data Availability

The data matrix and R script for the phylogenetic analyses are available in the Supporting Information. The original confocal and micro‐CT data are available in the Zenodo repository (https://doi.org/10.5281/zenodo.13996552).
